# Deploying the REACHnet Multi-State EHR-Based Network for Disease Surveillance (MENDS) node: lessons learned and tools utilized

**DOI:** 10.1093/jamiaopen/ooag166

**Published:** 2026-09-16

**Authors:** Sylvester Tumusiime, Lindsey Rudov, Kristina Larson, Kyle Bradford, Elizabeth Nauman, Meagan Alley, Bob Zambarano, Emily W Lankau, Amanda K Martinez, Katherine H Hohman, Thomas Carton

**Affiliations:** Health Services Research, Louisiana Public Health Institute, New Orleans, LA 70130, United States; Health Services Research, Louisiana Public Health Institute, New Orleans, LA 70130, United States; Health Services Research, Louisiana Public Health Institute, New Orleans, LA 70130, United States; Information and Technology Services, Louisiana Public Health Institute, New Orleans, LA 70130, United States; Health Services Research, Louisiana Public Health Institute, New Orleans, LA 70130, United States; Health Services Research, Louisiana Public Health Institute, New Orleans, LA 70130, United States; Healthcare Analytics, Commonwealth Informatics, a Qinecsa Solutions Company, Austin, TX 78731, United States; Emily W. Lankau Consulting, LLC, National Association of Chronic Disease Directors, Madison, WI 53705, United States; Iris Health Consulting, LLC, National Association of Chronic Disease Directors, Charlottesville, VA 22901, United States; Center for Public Health Leadership, National Association of Chronic Disease Directors, Atlanta, GA 30030, United States; Health Services Research, Louisiana Public Health Institute, New Orleans, LA 70130, United States

**Keywords:** REACHnet, public health, epidemiology, chronic disease surveillance, Multi-State EHR-Based Network for Disease Surveillance (MENDS)

## Abstract

**Objectives:**

We provide an overview of the transformation of REACHnet’s research-quality electronic health records (EHR) dataset into the Multi-State EHR-Based Network for Disease Surveillance (MENDS) dataset to support chronic disease surveillance.

**Materials and Methods:**

We highlight important governance and regulatory processes, infrastructure implementations, and partnerships that enabled this work, as well as the challenges faced.

**Results:**

REACHnet transformed clinical data from health systems in Louisiana and Texas into surveillance-ready datasets accessible to public health departments in Louisiana and Texas and the National Association of Chronic Disease Directors (NACDD).

**Discussion:**

Transforming reliably sourced and curated clinical data into a dataset that can be used by public health professionals for epidemiological surveillance activities required the development of governance, regulatory, and technical approaches that considered the priorities of all stakeholders. This work summarizes the challenges, opportunities, and approaches taken to develop a reliable and relatively low-latency dataset to support epidemiological studies by public health professionals.

**Conclusion:**

Our work underscores the importance of reliably sourced, standardized EHR data for chronic disease surveillance to inform public health policy and decision-making.

## Introduction

Electronic health records (EHRs) are widely used in clinical care^1^^,^^2^ and represent an important source of timely longitudinal data for public health surveillance.^3^ Whereas traditional public health surveillance activities have relied on state- and territorial-level managed surveys, disease registries, and vital records,^4^ interest has increased in modernizing existing surveillance systems to utilize growing data infrastructures.^5^ There is increasing evidence that robust, longitudinal EHR data are valuable for surveillance of chronic disease^2^^,^^3^ particularly in settings where timely and granular data are required for public health operations.

Chronic disease surveillance benefits from EHR data because standardized clinical domains such as demographics, vital signs, and encounter diagnoses support efficient case definitions development and longitudinal assessment over repeated healthcare encounters.^6^ Also, EHRs contain clinical measures such as blood pressure readings and body mass index that enhance condition detection and are often unavailable in claims-based surveillance data.^3^

Although EHR data have the potential for use in chronic disease surveillance, their operational utility remains contingent on data quality, completeness, and interoperability.^6^ Because only a subset of the population engages with healthcare services annually, and visit patterns may be sporadic, EHR-based surveillance data may be incomplete and not fully generalizable.^6^^,^^7^ These limitations are compounded by fragmented care across unconnected health systems, information-sharing barriers, and variability in clinical measurement practices that may affect accurate disease classification.^8–11^ In addition, patients receiving care across multiple facilities may contribute duplicate records, highlighting the importance of privacy-preserving de-duplication for the precision of estimates.^12^

Even with these limitations, EHRs can deliver useful clinical data for public health surveillance. The Centers for Disease Control and Prevention (CDC) piloted the Multi-State EHR-Based Network for Disease Surveillance (MENDS) in 2018 to build a network that leveraged existing EHR data models to support and enhance chronic disease surveillance efforts. MENDS operations are managed by the National Association of Chronic Disease Directors (NACDD).^13^ MENDS data are used by local and state health departments to inform program development, grant applications, and public health decisions and practice. For example, the New Orleans Department of Health used MENDS data to evaluate the prevalence of diabetes and hypertension across demographic groups and to inform program development and community outreach discussions.^14^

As of 2023, MENDS included 5 data contributors (ie, “nodes”), contributing data from approximately 91 health systems and 10 million patients in the United States. The first pilot partner to join the network was the Washington State Department of Health. MENDS became a multisite network when it gained additional data-contributing nodes, including the Research Action for Health Network (REACHnet). These new nodes enriched the MENDS data through additional and more diverse chronic disease cases and patient demographics.^15^

We provide an overview of key process components of REACHnet’s partnership with MENDS and illustrate the incorporation of a large, standardized clinical data resource into an evolving chronic disease surveillance system.

## Methods

### MENDS data sources and distribution

REACHnet collects and standardizes EHR data from health systems across California, Louisiana, and Texas using the PCORnet®, the National Patient-Centered Clinical Research Network Common Data Model (CDM)^16^ and creates research-quality datasets through quarterly data curation and quality assurance processes to support patient-centered research.^17^^,^^18^ Whereas the MENDS data infrastructure was built on the Electronic Medical Record Support for Public Health (ESP) database,^19^ REACHnet is built on the PCORnet® CDM. These data models were originally conceptualized for different purposes.

At the time of this analysis, REACHnet contributed data from 5 participating health system partners to MENDS, including 3 health systems in Louisiana and 2 in Texas. These systems represented a mix of large, multi-hospital integrated delivery systems and smaller community-based or specialty-focused health systems. Collectively, the participating health systems encompassed more than 100 hospitals and more than 1200 affiliated patient care centers, including inpatient hospitals, outpatient clinics, and specialty care facilities. Data contributed by these partners included both inpatient and outpatient encounters, enabling longitudinal capture of clinical data across diverse care settings. Participating sites were concentrated in metropolitan regions, including New Orleans, Dallas, Austin, and McAllen, Texas. Although not population-representative, the breadth of participating institutions and the inclusion of both inpatient and outpatient data provided a robust foundation for regional chronic disease surveillance analyses.

To assess population coverage after integration, linkage, and de-duplication, demographic characteristics of the MENDS population (aged 18 years or older) were compared with state-specific census estimates. Comparisons were conducted descriptively using absolute percentage-point differences. Hypothesis testing was not performed because census estimates represent fixed population distributions, and the REACHnet MENDS population constitutes a nonprobability sample of individuals seeking healthcare, limiting the interpretive value of statistical significance testing.

To characterize geographic distribution, patients were aggregated by zip code. Geographic visualizations were generated as choropleth maps using QGIS (QGIS Geographic Information System)^20^ with zip codes grouped into predefined bins to support interpretability and protect patient privacy.

### MENDS data governance and regulation

REACHnet’s Master Data Sharing and Use Agreement (MDSUA) with partner health systems permits the use of a Limited Data Set (LDS) consisting of standardized, de-identified, de-duplicated EHR data. On this basis, REACHnet entered into separate agreements with NACDD and participating Public Health Authorities (New Orleans Health Department (NOHD), Louisiana Department of Health (LDH) and the Texas Department of Health Services (DSHS)) to adapt the REACHnet CDM for public health activities. The data-sharing agreements with NACDD and participating Public Health Authorities were further augmented by data dissemination guidelines developed in partnership between REACHnet and NACDD. The guidelines are designed to preserve privacy by limiting the details exposed while reporting the prevalence of different chronic diseases by the Public Health Authorities.

### MENDS infrastructure development

Post regulatory approval, the MENDS infrastructure was modeled on the MDPHnet-distributed network for surveillance^21^ requiring a multidisciplinary team to scale the existing MDPHnet network to a national level. The operational considerations, including deployment timelines, staffing requirements, and ongoing technical maintenance, are described in prior MENDS publications and varied across sites based on local infrastructure, data readiness, and capacity.^21^^,^^22^ Establishing the REACHnet MENDS node from initial deployment through to Stage 2 validation took nearly 5 months and required extensive coordination between a full-time-equivalent technician and internal specialists to identify and deploy the hardware components and configuration necessary to meet the robust specifications required for the MENDS infrastructure. Ongoing maintenance by the technology vendor is on the order of 100 hours per year.

MENDS operates through partnerships with 5 data-contributing networks, with REACHnet serving as a key partner covering Louisiana and Texas. This partnership leverages REACHnet’s PCORnet-standardized data to provide MENDS with research-grade clinical information for public health surveillance. The underlying data model for MENDS query tools is the open-source ESP for automated chronic disease identification from EHR data.^19^

Under the existing MDSUAs governing data use, data from participating REACHnet health system partners stored in the PCORnet CDM^23^ format were merged to generate the MENDS “Combined Common Data Model” (CCDM). The CCDM leverages a unique privacy-preserving record linkage (PPRL)^23^ software suite from Datavant, to deduplicate patients and harmonize a distinct patient’s clinical record from different health systems into records that belong to 1 distinct patient identifier. To perform the merge, the Datavant Connect suite^24^^,^^25^ was used, involving 2 stages of data transformation, including: (1) tokenization^24^ and (2) matching to link patient records within and between health systems.^25^ The default setting, which balances precision (fraction of accurate matches) and recall (the representation of all possible matches), was used.

### MENDS data validation

A 4-stage data validation ensured that the data ingested into the MENDS datamarts were of high quality and reproducible:

1. Comparisons between source and ESP databases: A high-level comparison between source and ESP datasets for expected vs observed values was performed by a single person followed by a combined effort between the REACHnet and NACDD teams. Metrics included: patient counts, number of encounters, comparison of date ranges, and counts for gender, race, and ethnicity. This analysis was performed by the Commonwealth Informatics Inc. (CII) team.
2. Statistical summarization of the data: The high-level comparison was followed by an extensive statistical summarization of the data via a set of listings, tabulations, and cross-tabulations using custom scripts^26^ by the CII and NACDD teams. This allowed application of rules for data exclusions, such as systolic blood pressure values above 300.
3. Algorithmic phenotype identification: Identification of a set of disease conditions, such as essential hypertension or type 2 diabetes, followed the statistical summarization. This involved the CII team randomly selecting 20 patients for each condition and generating a list of their data, including associated relevant medical events such as laboratory test results. REACHnet staff then reviewed the listings against the source data for accuracy.
4. External algorithmic phenotype validation: Finally, we generated prevalence statistics for the disease conditions of interest and compared the results against external data sources including the Behavioral Risk Factor Surveillance System (BRFSS), the National Health and Nutrition Examination Survey (NHANES), and MDPHnet^27–29^ for the same phenotypes. These external sources were used because they provide well-established, complementary benchmarks for chronic disease prevalence that align with the conditions captured in MENDS.

### MENDS data weighting and modeling

Because the source data used for MENDS do not represent the total population of the geographic region, and because partner sites were not composed of a random sample, MENDS data were not fully representative of the United States or local sociodemographic distributions. Instead, the data represented a sample of patients seeking care.

To address this issue, MENDS applied systematic weighting and modeling approaches to generate more population-aligned prevalence estimates. In collaboration with NORC at the University of Chicago (NORC),^30^ MENDS used post-stratification weighting^31^ to align EHR data with national population benchmarks, enabling comparison with established surveillance systems such as NHANES. This approach enabled assessment of whether EHR-based surveillance could produce credible prevalence estimates to support ongoing chronic disease surveillance efforts. After joining the MENDS network, REACHnet implemented the post-stratification weighting approach for hypertension prevalence and hypertension control analyses using EHR data from approximately 800 000 adults, aggregated at the state and parish (count-equivalent) level.

### MENDS data analysis

**Figure 1.**
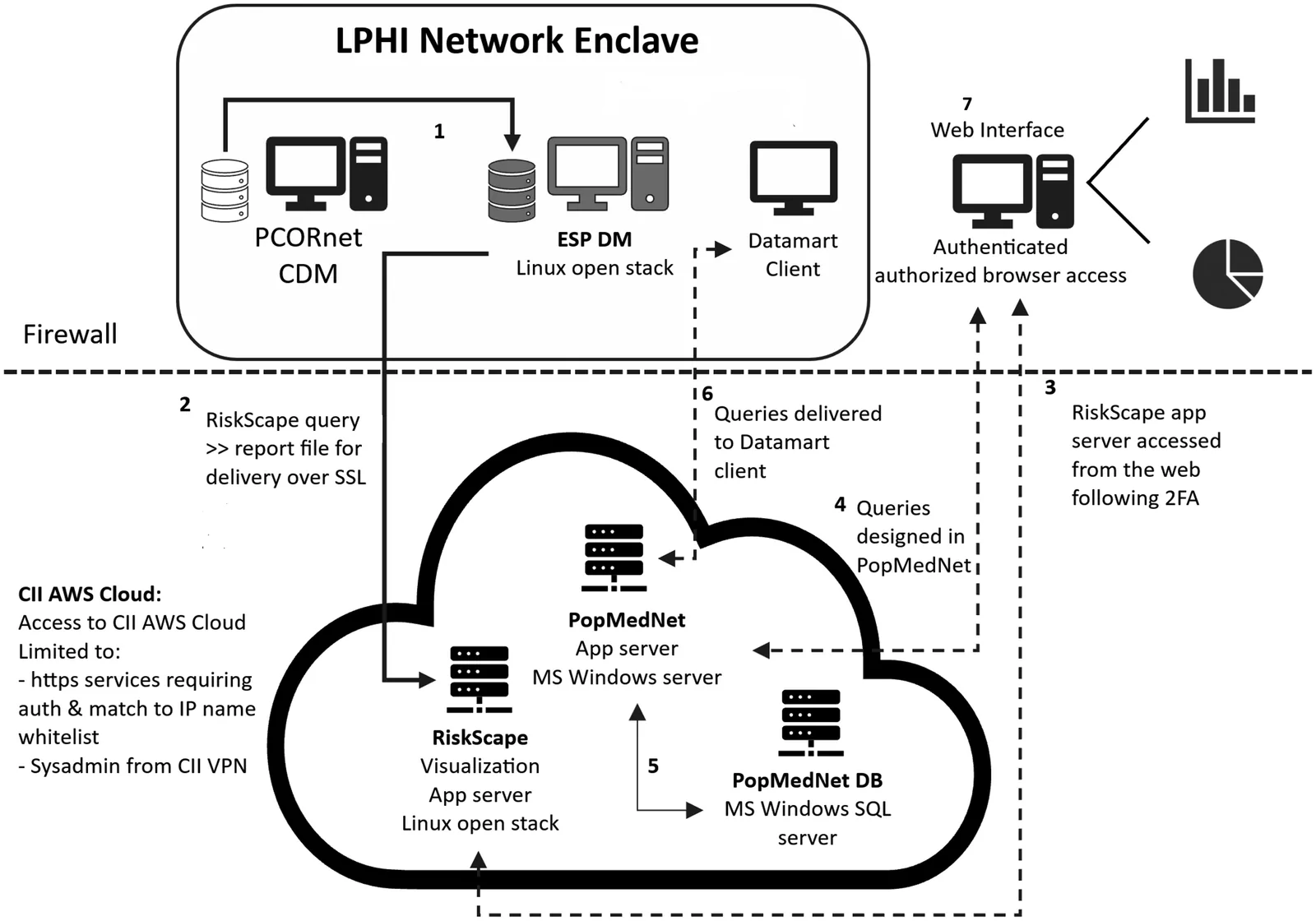
The MENDS data flow: (1) Data were extracted from the parent datamart (PCORnet CDM) and delivered to the MENDS ESP datamart. (2) The ESP data were formatted to the RiskScape specification to support visualization in the RiskScape application. (3) Data users accessed the RiskScape data visualization server via a web interface (7). Data access was also achieved via queries designed in the PopMedNet App server (4) over a web interface (7) against the MENDS PopMedNet database (5). The PopMedNet queries and results were delivered (6) to a Datamart Client from which aggregate data were retrieved.

To support disease surveillance and analysis, MENDS implemented 2 complementary data access platforms. The RiskScape platform (Figure 1 (7)) provides interactive, web-based access to de-identified data stored in the ESP database, enabling exploratory visualization of disease prevalence and trends.^32^ Simple queries could be run on the RiskScape web-based data visualization tool by defining a cohort using drop-down fields to define the population(s) of interest. In parallel, the PopMedNet platform (Figure 1 (5)) supported complex interrogation of data. Approved data users submitted queries against the MENDS database using PopMedNet^33^ which enabled development and distribution of queries targeting populations and conditions of interest. A self-service system was also developed by NACDD,^13^ to enable MENDS data querying via archived prevalidated custom queries hosted on GitHub. The prevalidated custom queries had built-in controls that enforced the MENDS MDSUAs. All MENDS data were subject to the safe harbor method of de-identification through Datavant’s tokenization algorithm.^24^ Data could alternatively be reviewed by experts as part of the de-identification process.

## Results

### Patient overlap results

**Figure 2.**
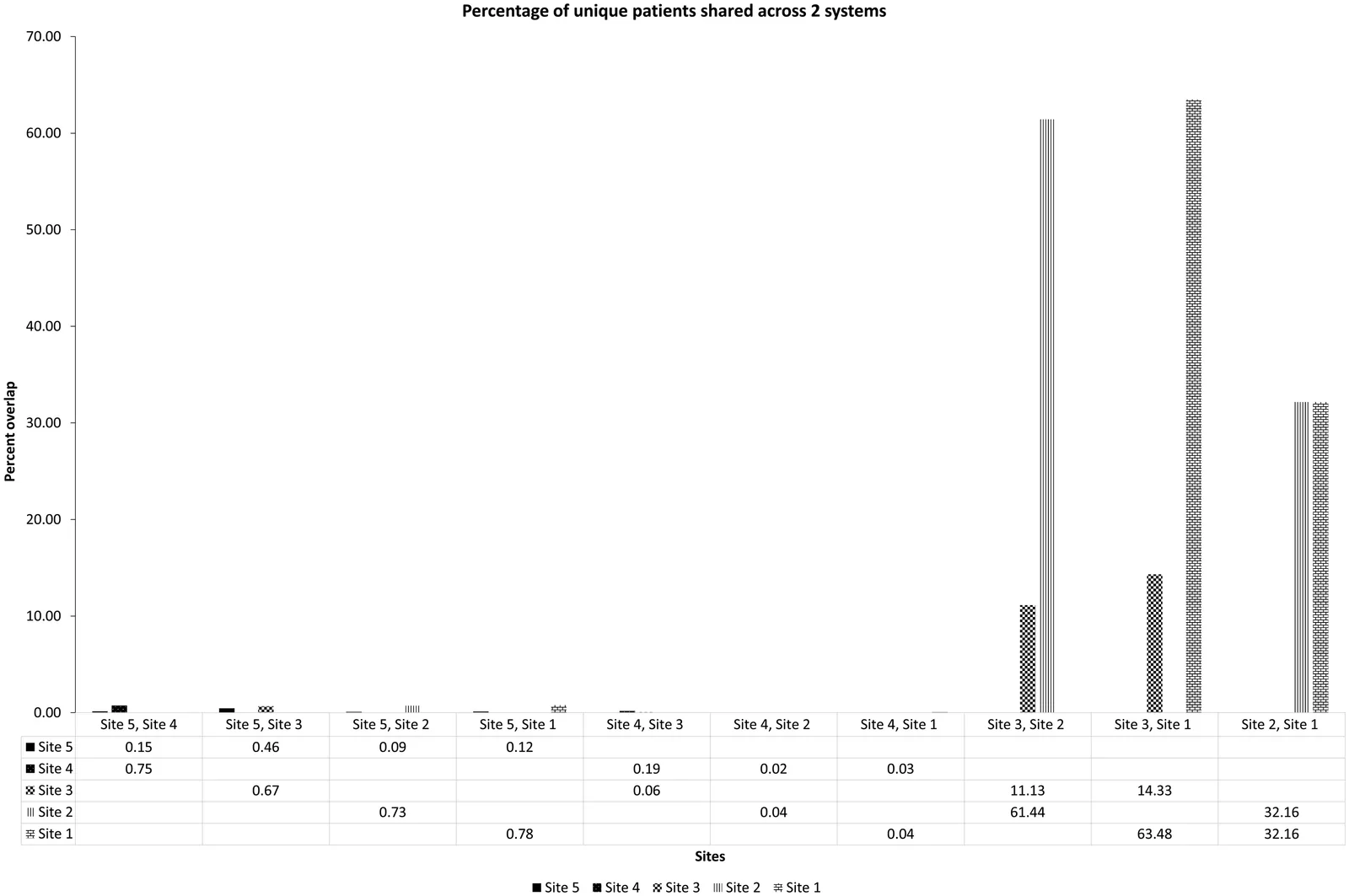
Percentage of unique patients shared across 2 systems—October 2024. The data were generated by evaluating tokenized patient data using the Datavant match algorithm across 2 health systems.

Results are based on the October 2024 MENDS CCDM, consisting of data from 5 participating health system partners across Louisiana and Texas. The population analyzed was composed of de-duplicated patients with at least 1 qualifying inpatient or outpatient encounter since February 1, 2018. Analyses were restricted to patients aged 18 years and older to align with chronic disease surveillance indicators and public health reporting objectives. An overlap assessment was performed to identify unique patients with encounters recorded at 2, 3, or 4 health system partner sites. Within geographically proximate locations within the same state, approximately 30%-60% of patients from smaller health systems were represented in the larger health system, whereas overlap in the reverse direction was substantially lower (11%-14%; Figure 2). This could be a function of larger systems having a larger geographical footprint of combined discrete patient care centers thereby increasing the probability of encounters. An approximation of the patient care centers for each health system (Table 1) when compared against the percentage of unique patients in each pair of health systems (Figure 2) supports the observation.

**Table 1.** Number of hospitals and patient care centers per affiliate health system.

Health system partner site	Number of hospitals	Number of patient care centers
Site 1 (LA, small)	1	1
Site 2 (LA, small)	1	1
Site 3 (LA, large)	46	370
Site 4 (TX, small)	1	60 (Specialty Centers)
Site 5 (TX, large)	52	800

**Figure 3.**
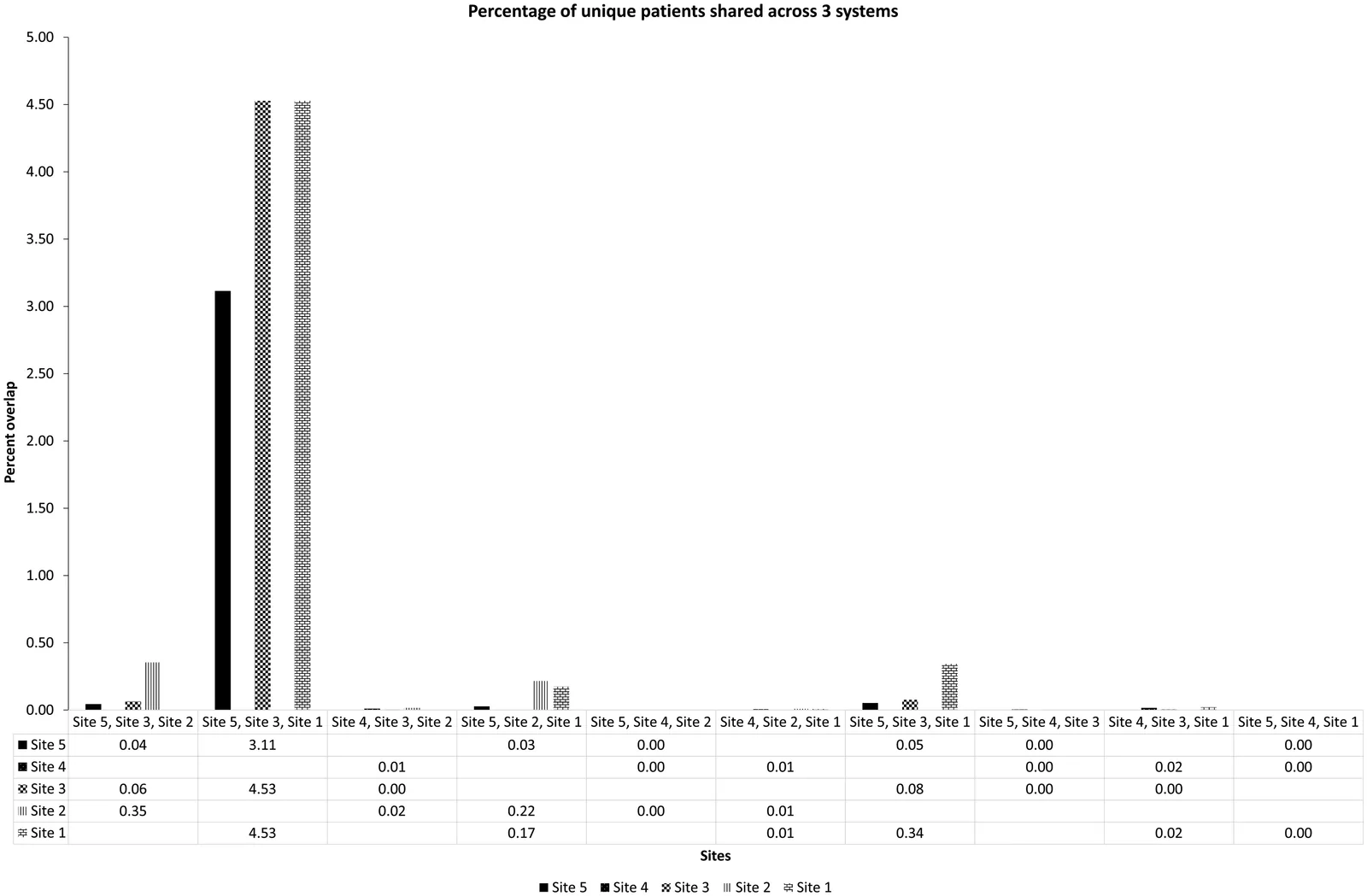
Percentage of unique patients shared across 3 systems—October 2024. The data were generated by evaluating tokenized patient data using the Datavant matching software across 3 health systems.

**Figure 4.**
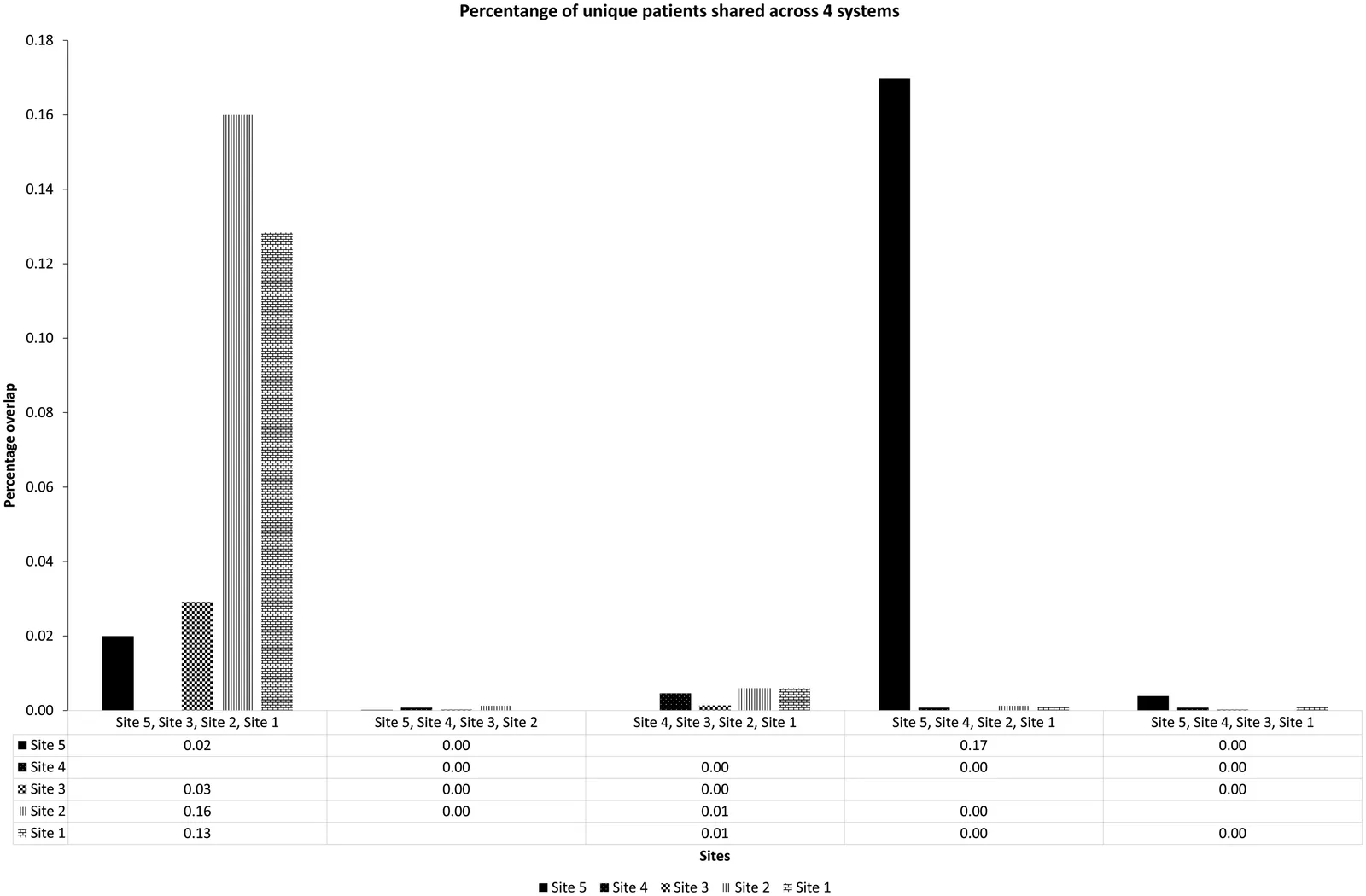
Percentage of unique shared patients across 4 systems—October 2024. The data were generated by evaluating tokenized patient data using the Datavant matching software across 3 health systems.

**Figure 5.**
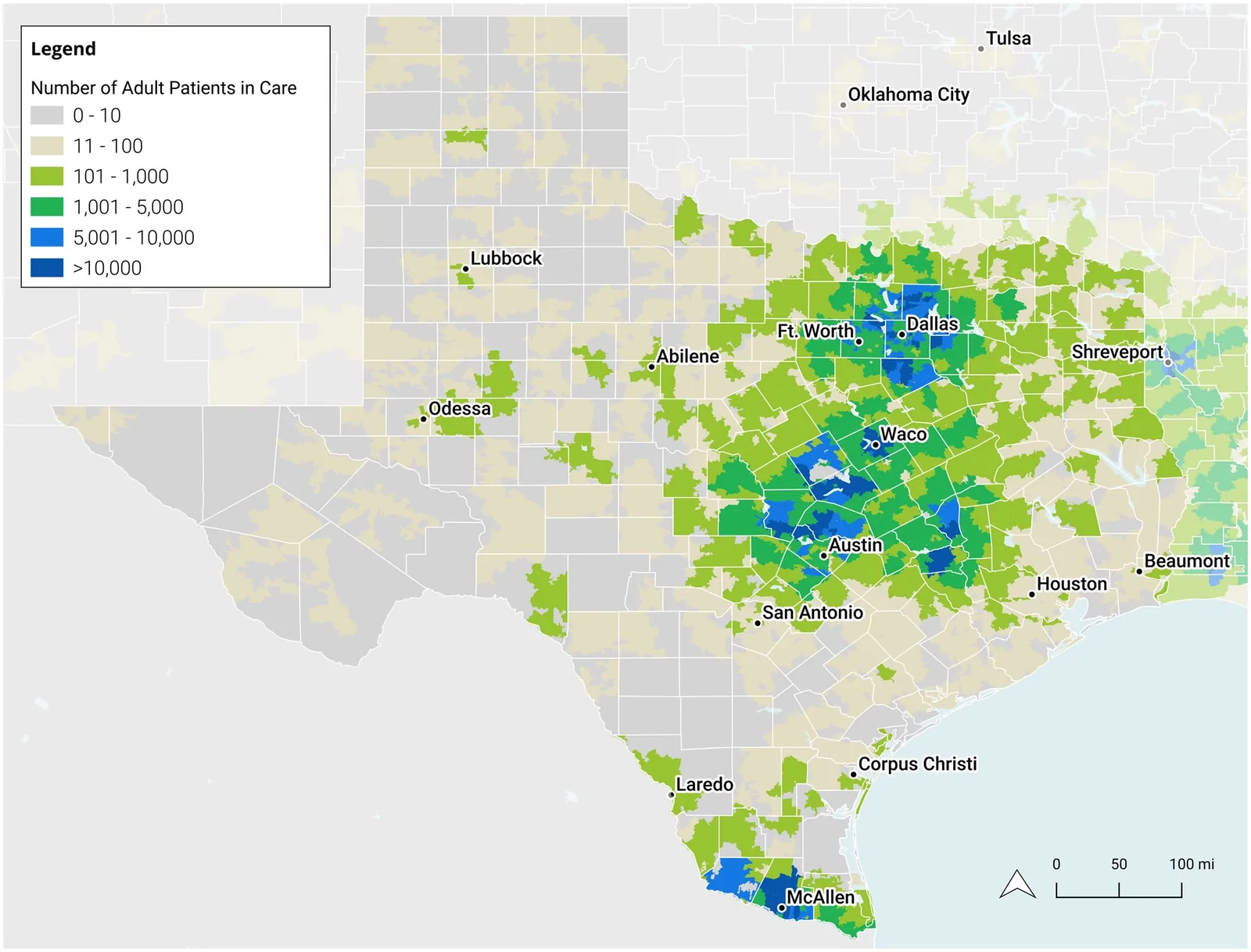
Number of adult patients in care in the Multi-State EHR-Based Network for Disease Surveillance (MENDS), by ZIP code—Texas, 2024.

**Figure 6.**
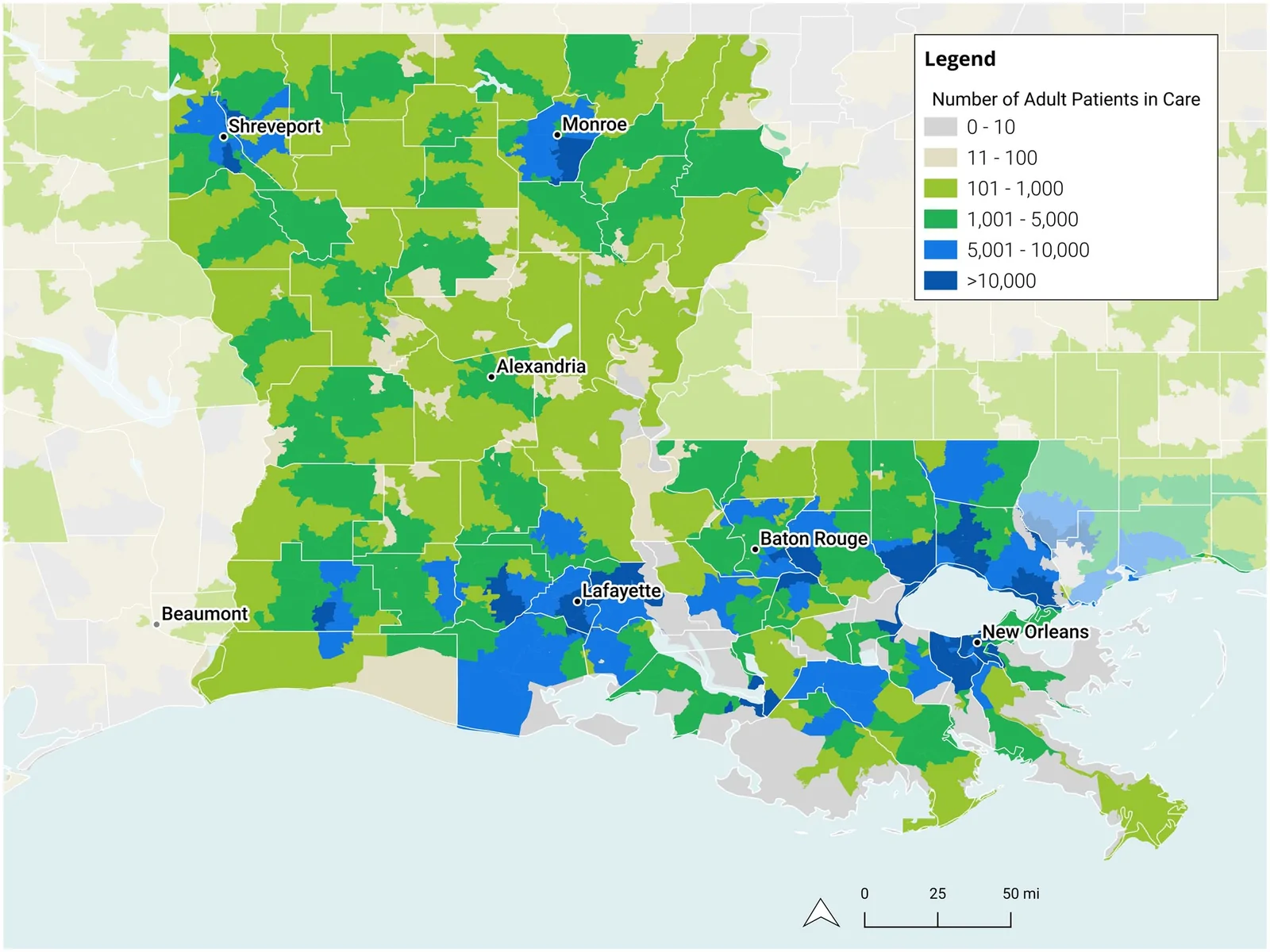
Number of adult patients in care in the Multi-State EHR-Based Network for Disease Surveillance (MENDS), by ZIP code—Louisiana, 2024.

An exception to the general overlap pattern was observed between Site 4 and Site 5, where the overlap between the systems ranged from 0.15% to 0.75%, likely due to minimal geographic overlap in care delivery regions (Austin/Dallas vs Southern Texas). Overlap percentages decreased substantially across 3 (Figure 3) and 4 (Figure 4) partner health systems. The geographic distribution of adult patients in care across REACHnet MENDS participating systems is shown in Figures 5 and 6, illustrating the concentration of care within distinct metropolitan regions in Texas (Figure 5).

### Patient sex distribution in REACHnet MENDS node relative to government records

Understanding the demographic profile of a given community is important given the unique health needs associated with sex, age and race and ethnicity. Therefore, we compared the demographic characteristics of the REACHnet MENDS population with state-specific US census estimates^34–37^ to assess alignment between the MENDS population and the underlying populations in Louisiana and Texas (Table 2). Table 2 presents the state-stratified distributions of age, sex, and race and ethnicity, along with the percentage-point differences between the MENDS population and the corresponding state census estimates.

**Table 2.** State-specific distribution and percentage-point differences in sex, race/ethnicity, and age among adults aged ≥18 years in MENDS CCDM compared with state-specific 2020 Census populations.

Characteristic	Louisiana MENDS population (%)	Louisiana census population (%)	Louisiana percentage-point difference (Census-MENDS)	Texas MENDS population (%)	Texas census population (%)	Texas percentage-point difference (Census-MENDS)
**Sex**						
Female	55.4%	52.2%	−3.2%	57.0%	51.2%	−5.9%
Male	44.6%	47.8%	3.2%	42.9%	48.8%	5.9%
**Race**						
American Indian or Alaska native	0.3%	0.5%	0.2%	0.3%	0.3%	0.1%
Asian	1.4%	1.9%	0.5%	4.2%	5.5%	1.3%
Black or African American	31.9%	29.9%	−2.0%	12.3%	11.8%	−0.5%
Hispanic or Latino	5.8%	6.3%	0.5%	26.5%	36.2%	9.7%
Multiple Race	0.0%	2.7%	2.7%	1.1%	2.6%	1.5%
Native Hawaiian or Other Pacific Islander	0.0%	0.0%	0.0%	0.2%	0.1%	−0.1%
Other	1.3%	0.3%	−1.0%	1.0%	0.4%	−0.6%
White	55.3%	58.3%	3.0%	48.3%	43.2%	−5.1%
Refused/Missing/Unknown	4.0%	0.0%	−4.0%	6.3%	0.0%	−6.3%
**Age (years)**						
18-44	43.7%	46.1%	2.4%	42.1%	50.2%	8.1%
45-64	30.1%	32.5%	2.4%	30.7%	31.9%	1.2%
65+	26.2%	21.4%	−4.9%	27.2%	17.9%	−9.3%

Percentages are calculated using adult populations aged ≥18 years. Census estimates are derived from state-specific 2020 Decennial Census Demographic and Housing Characteristics data for Louisiana and Texas.^38^ Race and ethnicity were harmonized such that individuals identified as Hispanic or Latino were classified as Hispanic regardless of race, and non-Hispanic individuals were classified by reported race. Percentage-point differences reflect census minus MENDS value within each state.

The female population composed a higher proportion of the REACHnet MENDS population, tracking with census records for females in Louisiana and Texas (Table 2). Overall, the percentage population of the different sexes captured by the REACHnet node relative to census data were comparable (Table 2).

### Patient race distribution in REACHnet MENDS node relative to census data

In Louisiana, White and Black individuals comprised the largest race/ethnicity groups within the REACHnet MENDS population, followed by Hispanic or Latino individuals (Table 2). Relative to Louisiana census estimates for adults aged 18 years or older, the MENDS population included a slightly higher proportion of Black individuals (31.9% vs 29.9%) and a slightly lower proportion of White individuals (55.3% vs 58.3%). The proportion of Hispanic or Latino adults in the Louisiana MENDS population was similar to the census estimates.

In Texas, Hispanic or Latino, White, and Black individuals accounted for most of the adult population in both MENDS and the Texas census data. Compared with Texas Census estimates, the Texas MENDS population included a substantially lower proportion of Hispanic or Latino individuals (26.5% vs 36.2%) and a higher proportion of White individuals (48.3% vs 43.2%). Differences for Black individuals were small (12.3% in REACHnet MENDS vs 11.8% in Texas census data). Across both states, smaller race/ethnicity categories differed by only a few percentage points.

### Patient age distribution in REACHnet MENDS node relative to census data

The age distribution of individuals in the REACHnet MENDS population differed from state-specific census estimates in both Louisiana and Texas (Table 2). In Louisiana, individuals aged 65 years and older comprised a larger proportion of the MENDS population compared with the census data (26.2% in REACHnet MENDS vs 21.4% in Louisiana Census data). Individuals aged 18-44 were modestly underrepresented (43.7% vs 46.1%). In Texas, similar patterns were observed, with adults age 65 years and older representing a larger proportion of the REACHnet MENDS population relative to census estimates (27.2% vs 17.9%), and adults aged 18-44 years representing a smaller proportion (42.1% vs 50.2%).

### Geographic distribution of patients in care in the REACHnet MENDS node

We evaluated the distribution of adult patients in care at participating health systems within the REACHnet MENDS node. The Texas patient population was concentrated in the Dallas, Waco, and Austin areas, as well as the McAllen area on the southern tip of the state (Figure 5). In Louisiana, the distribution of patients in care was not as acutely aggregated as the distribution of patients in Texas, with fewer areas in Louisiana having 10 or fewer patients associated with the health systems participating in MENDS (Figure 6). However, even in Louisiana, coverage was higher around metropolitan areas than in highly rural parts of the state.

### Data weighting and modeling example from the REACHnet MENDS node

The weighted hypertension prevalence estimates derived from the weighting and modeling approach were approximately 10% lower than the crude estimates and approximately 13% higher than the hypertension awareness estimates from the 2021 BRFSS across a similar geographic and demographic profile. These data show that post-stratification weighting was able to bring the EHR-based data estimates closer to the survey-based estimates and is a useful tool for aligning near real-time crude clinical EHR data available in MENDS with the required standards necessary for accurate reporting by public health organizations.^31^

## Discussion

This work presents a scalable informatics framework for repurposing research-oriented CDM data nationally across PCORnet CDM sites, into a surveillance-ready, multi-jurisdictional chronic disease monitoring system through harmonized governance, privacy-preserving record linkage, and population-level calibration. The tools developed for MENDS have enabled public health agencies to assess chronic disease patterns using relatively more recent data than those available through survey-based approaches (ie, BRFSS and NHANES) and at a finer geographic resolution than is typically available. For example, health departments have used RiskScape to examine hypertension prevalence across urban neighborhoods, while PopMedNet has supported tailored analytic requests through validated query workflows.

### Limitations

An important consideration when interpreting findings derived from MENDS is the inherent variability in data quality, completeness, and clinical practice patterns across contributing health systems. Participating sites differ in size, geographic footprint, patient populations served, and care delivery models, which can influence encounter frequency, documentation practices, and the availability of specific clinical elements. For example, outpatient-focused systems may contribute richer longitudinal data for chronic disease management indicators such as blood pressure or laboratory monitoring, whereas inpatient-heavy systems may more strongly capture acute events and hospital-based diagnoses. These differences can introduce heterogeneity into EHR-based surveillance estimates, particularly when aggregating data across multiple systems and regions.

In addition, EHR-based surveillance is subject to several well-recognized sources of bias. Clinical data reflect individuals who interact with the healthcare system and therefore may underrepresent populations with limited access to care or lower healthcare utilization. Variability in visit frequency, diagnostic coding practices, and measurement protocols across sites can further influence disease ascertainment and prevalence estimates. The MENDS infrastructure addressed some of these challenges through standardized data harmonization using the PCORnet CDM, systematic data quality assessment, privacy-preserving patient linkage to reduce duplicate records, and post-stratification weighting to align EHR-derived estimates with census-based population characteristics. However, these approaches mitigate rather than eliminate bias, and residual differences between EHR-based and survey-based estimates should be expected. As such, MENDS data and other similar approaches are best interpreted as complementary to traditional public health surveillance systems, offering enhanced timeliness and geographic resolution while requiring careful contextualization of the underlying data sources. Therefore, a hybrid approach that uses both EHR- and non-EHR analysis is recommended for comprehensive chronic disease surveillance.

### Lessons learned

Data processing introduces latency: There is a lack of real-time data because of the time needed to transform EHR data into a usable dataset in the MENDS and RiskScape datamarts (3-4 months). EHR-to-MENDS data transformation is a complex and lengthy stepwise process occurring quarterly and requires collaboration between REACHnet data analysts and various health system partners to ensure conformance of EHR data to the PCORnet CDM specification^39^ and ultimately its conversion to a singular MENDS database. The associated latency does not significantly affect clinical research studies but could have a negative impact on the ability to rapidly evaluate EHR data for emerging or evolving infectious diseases over shorter timeframes. However, it remains valuable to participating health departments for chronic disease surveillance.^14^ Even with this inherent latency, the MENDS approach is relatively rapid when compared with traditional surveillance timelines which approximate multi-year lags between data collection and publication.^40^ Furthermore, traditional surveillance tools, such as BRFSS, can lack pediatric data, data belonging to the populations without telephones, or data for smaller cities and towns, and have limited capacity to estimate the prevalence of rare conditions.^40^

Skewed coverage of EHR data: EHR population coverage of patients receiving care in participating hospitals and health systems in the REACHnet system is not random in Texas or Louisiana (see Figures 5 and 6, which show higher patient density in several metropolitan areas). This can result in biased estimates at both the state and county levels, requiring statistical methods (eg, post-stratification weighting based on census-based population) to align observations with underlying population levels.^31^ Differences between the MENDS data node and census data with respect to some races (Table 2) might contribute to the observed crude estimates, thereby supporting the need for modeled estimates of chronic disease.

User experience is vital when developing surveillance tools: The targeted querying of the MENDS/ESP databases via the PopMedNet tool requires an in-depth understanding of the ESP schema to extract meaningful results that are actionable by public health department staff and other users. PopMedNet provides an alternative to the RiskScape platform because it enables generation of aggregate data in a downloadable format vs the RiskScape platform, which is geared to graphical representation of the MENDS data. To minimize the query inefficiencies arising from the complexity of MENDS databases, a modular approach to PopMedNet queries that uses previously validated queries stored in the MENDS GitHub repository. Public health users can use validated queries by replacing relevant code lists and demographics to match their interests. This evolution illustrates the adaptability of the system to increased query volumes.

Although users have reported value in the ability to generate map visualizations and graphs using the RiskScape platform, the lack of the capacity to visualize data by parish or zip code has been reported as a limitation of the platform. This underscores the necessity to balance user needs with regulatory compliance checks that necessitate inhibiting some functionality.

### Conclusions and future directions

We provide a broad overview of the transformation of a comparative effectiveness research clinical database into a tool that can be used for actionable disease surveillance. Different considerations, such as governance and compliance, infrastructure establishment, as well as data normalization, that are necessary for the development of a health system-based surveillance tool, are summarized.

The ultimate value of the MENDS platform–the culmination of efforts on governance, infrastructure, standardization, and visualization–is “data for action.” The MENDS platform is regularly and actively utilized by public health authorities to answer questions of importance to their programming, populations, and policies. Regular monthly meetings between public health practitioners and MENDS technical experts are used to co-develop questions of interest, methods and algorithms to answer those questions, and key extensions of the program through original de novo queries of the data. This actionability is the impact, leveraging MENDS to answer real-life problems in real-world settings.

This overall impact can be improved with more timely data for faster real-time action for public health officials. Therefore, strategies that can reduce data latency need to be considered and integrated. FHIR (Fast Healthcare Interoperability Resources) is a specification for healthcare interoperability that uses modern web technologies such as RESTful APIs to enable secure, granular, and real-time patient data exchange between health systems.^41^ FHIR uses “Resources” to abstract common clinical concepts and has been through iterative cycles of improvement, leading to the latest version (5.0.0).^42^ FHIR APIs have been used to support automated, compliant public health reporting using clinical data^43^ and therefore are candidates for decreasing latency by directly linking health system EHRs with disease surveillance tools. As regulatory demands expand the mandates for interoperability between health systems, the importance of tools such as FHIR and other technologies in the delivery of timely and actionable surveillance data will likely increase, resulting in improved surveillance systems.

## Data Availability

The data underlying this article are available in the article.
